# Protocols for Prone Positioning in Pediatric Patients with Hypoxemia: Impact on Oxygenation, Lung Function, and Clinical Safety

**DOI:** 10.3390/children12060743

**Published:** 2025-06-07

**Authors:** Jose Luis Estela-Zape, Valeria Sanclemente-Cardoza, Leidy Tatiana Ordoñez-Mora

**Affiliations:** 1 Faculty of Health, Universidad Santiago de Cali, Cali 760035, Colombia; leidy.ordonez01@usc.edu.co; 2 Health and Movement Research Group, Universidad Santiago de Cali, Cali 760035, Colombia; 3 Faculty of Health, School of Public Health, Universidad del Valle, Cali 760043, Colombia; valeriasanclemente0@gmail.com

**Keywords:** prone position, pediatrics, protocols, artificial respiration, lung injury

## Abstract

**Background/Objectives**: This review aims to identify existing protocols and evaluate the effects of prone positioning on oxygenation and clinical outcomes in pediatric patients with hypoxemia. **Methods**: A systematic review was conducted in accordance with the PRISMA guidelines and registered in PROSPERO (CRD42023457270). Literature research was performed in Scopus, PubMed, Web of Science, and ScienceDirect. The final search was completed in January 2025. **Results**: A total of 2033 studies were identified, with 5 meeting inclusion criteria. Forty percent applied prone positioning for 12 to 20 h, improving pulmonary function. Combined with alveolar recruitment, prone positioning increased functional residual capacity and reduced atelectasis, with SpO_2_ improvements from 13% to 38% and atelectasis reduction from 8% to 47%. Another 40% focused on oxygenation, reporting PaO_2_ increases from 52 to 59 mmHg and SpO_2_ improvements from 93.2% to 96.2% within 2 to 4 h. One study found a significant SpO_2_ difference between prone (98.3%) and supine (96.2%) positions (*p* = 0.003). Protocols commonly included facial tilt and pillows to reduce compression. Safety measures involved checking catheter and tube placement, suspending enteral nutrition 30 min before repositioning, and hemodynamic monitoring. Adverse events were rare, including two cases of tube obstruction and one hypercapnia. No significant differences were observed in ventilation duration, oxygen therapy length, or 28-day survival between groups. **Conclusions**: Prone positioning improves pulmonary function and addresses refractory hypoxemia in pediatric patients. However, the optimal duration remains unclear, underscoring the need for further research to establish standardized guidelines.

## 1. Introduction

Prone positioning is commonly used in the management of pediatric patients requiring invasive mechanical ventilation due to pulmonary dysfunction and severe hypoxemia [1]. This approach reduces the pleural pressure gradient, generating more negative dorsal pleural pressures, increasing transpulmonary pressure, and improving alveolar permeability in patients with severe lung diseases [2,3]. It also induces key physiological changes, such as increased functional residual capacity via alveolar recruitment, lung re-expansion, improved ventilation–perfusion ratio (V/Q), reduction in pulmonary shunt, and optimization of positive end-expiratory pressure [4,5].

Prone ventilation is particularly effective for patients with refractory hypoxemia (PaO_2_ ≤ 60 mmHg) [6], resulting from conditions including pneumonia, pediatric acute respiratory distress syndrome (ARDS), hyaline membrane disease, pulmonary hypertension in neonates, congestive heart failure, and respiratory failure following cardiac surgery [7,8]. ARDS affects 85.4% of premature newborns (<28 weeks) and 44.1% of those born at 28–32 weeks gestation. It is the most prevalent condition in this population and is associated with approximately 40,000 cases annually worldwide, representing the leading cause of neonatal death within the first week of life [4]. However, no standardized ventilatory protocols for prone positioning exist for pediatric patients with refractory hypoxemia. Studies in adults, such as the PROSEVA trial [9], suggest that prone positioning improves V/Q and oxygenation and reduces mortality in severe ARDS. Prolonged prone positioning (>72 h) [10] and early initiation in adults have been shown to decrease mortality at 28 and 90 days [6]. Pediatric studies [11,12] report similar outcomes, including enhanced lung compliance and reduced airway resistance [13,14].

The implementation of the PROSEVA protocol in pediatric intensive care units (PICUs) presents challenges due to anatomical and physiological differences between children and adults [15]. The lack of pediatric-specific prone positioning protocols and limited evidence regarding their effectiveness highlight the need to adapt these strategies to the unique characteristics of pediatric patients. Further research is necessary to assess the safety and efficacy of prone positioning in this population to support evidence-based recommendations [16,17].

The optimal duration of prone positioning in pediatric patients with hypoxemia remains undefined [14,18]. However, advanced pulmonary and thoracic monitoring techniques, such as electrical impedance tomography and biomarker analysis, offer precise tools to guide the timing of transitions between supine and prone positions [19]. Given the limited data on the use of prone positioning during mechanical ventilation in pediatric patients with hypoxemia, this review aims to consolidate current evidence to better understand its effectiveness, safety, and potential benefits or risks.

The primary objective of this review is to identify existing protocols and evaluate the effects of prone positioning on oxygenation, lung function, and clinical safety outcomes in pediatric patients with hypoxemia.

## 2. Materials and Methods

A systematic review was conducted following the guidelines outlined in the PRISMA statement [20], with registration in PROSPERO (CRD42023457270).

### 2.1. Research Question

The research question was formulated using the PICO framework: What are the protocols and effects of prone positioning during invasive mechanical ventilation in pediatric patients with hypoxemia? The question was designed based on the following criteria for population, intervention, and outcomes:

- P (Population): Pediatric patients with artificial airways and hypoxemia.
- I (Intervention): Prone positioning during invasive mechanical ventilation.
- C (Comparison): Not applicable.
- O (Outcome): Prone positioning protocols, effects on oxygenation, reduction in lung injury, and associated complications.

### 2.2. Search Strategy

Based on the research question, a comprehensive search was conducted in the following electronic databases: Web of Science, PubMed, Scopus, and ScienceDirect. The search terms were determined using Medical Subject Headings (MeSH), Health Sciences Descriptors (DeCS), and natural language.

#### Search Terms

This review was based on existing studies that addressed the previously described topic, including management protocols in the pediatric population. The selection of studies was limited to the PICO question using the following search terms: “pediatrics,” “children,” “prone position,” “artificial ventilation,” and “protocols.” The logical operators “AND” and “OR” were used, which allowed us to generate the equation.

### 2.3. Eligibility Criteria

The following selection and inclusion criteria were applied: articles addressing the review topic, “Protocols and effects of prone positioning during invasive mechanical ventilation in pediatric patients with hypoxemia,” and articles detailing protocols related to the duration of prone positioning and transitions between supine and prone positions during mechanical ventilation. Only studies involving pediatric patients aged 0 to 18 years (infants/toddlers, school-age children, adolescents) with artificial airways and hypoxemia were included, and publications were accepted without language restrictions. Exclusion criteria included studies where the population did not have artificial airways or receive supplemental oxygen support, as well as secondary literature, such as systematic reviews, reflective reviews, conference proceedings, theses, partial data, and letters to the editor.

### 2.4. Selection of Sources of Evidence

A systematic search was conducted across multiple databases to identify relevant studies. Duplicates were subsequently removed to maintain the integrity of the selection process. Each study was independently assessed based on predefined inclusion criteria, and those that did not meet these criteria or were duplicated across sources were excluded. To ensure the reliability of the study selection, an agreement analysis between reviewers was performed. Any discrepancies were resolved through discussion or, when necessary, by the involvement of a third reviewer, ensuring accuracy and objectivity in the final selection. The included studies specifically examined the effects and efficacy of supplemental oxygen administration in the prone position, without considering the underlying pathology or the specific indications for its use (Appendix A).

### 2.5. Data Extraction

A descriptive table was created in Excel once the review and selection of the articles that met the inclusion criteria was completed, and relevant data for each article was collected, including information about the author, year of publication, country where the study was carried out, sample size, study design, and detail of the intervention protocol used. The effects detected in aspects such as oxygenation, lung function, reduction in lung lesions, and possible complications during mechanical ventilation in the prone position in pediatric patients were also included.

### 2.6. Data Synthesis

The selected articles, which met the eligibility criteria, provided crucial data on the duration and sequence of prone and supine positions and the benefits associated with prone positioning in severe lung diseases. The collected information was organized into descriptive tables highlighting the concepts relevant to the investigation. A flowchart was also outlined based on the PRISMA statement guidelines [20], illustrating the search and selection process for the included studies.

### 2.7. Quality Assessment

The quality of the studies was assessed independently and in a blinded manner using the PEDro scale [21]. The PEDro scale was employed to evaluate the quality of randomized controlled trials, focusing on factors such as participant randomization, allocation concealment, blinding in assessments, and the use of standardized outcome measurement methods.

## 3. Results

**Figure 1:**
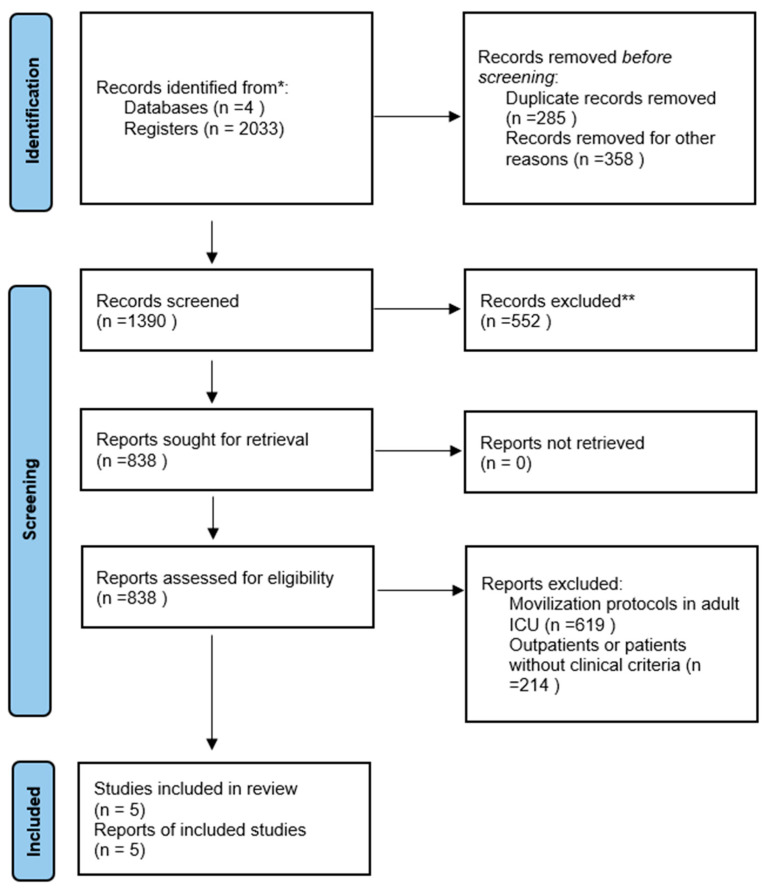
Flowchart of the systematic review. * Web of Science, PubMed, Scopus, and ScienceDirect ** Review by title.

The initial search identified 2033 studies across four databases. After removing duplicates and articles for various reasons, 1390 records were screened for eligibility, resulting in the selection of 5 articles for full review and methodological quality assessment. Figure 1 provides a graphical representation of the study selection process.

### 3.1. Methodological Quality Assessment

The studies selected, published between 2001 and 2020, employed a randomized controlled methodology. After applying the PEDro scale [21], the studies achieved scores ranging from six to eight points, as shown in Table 1.

### 3.2. Effect of Prone Positioning on Pulmonary Function and Oxygenation

The review of studies showed that 40% employed prone positioning to enhance lung function in conditions like acute lung injury, with positioning maintained for 12–20 h over a span of 7 days [22,25]. Additionally, Eun et al. (2020) [26] found that combining prone positioning with alveolar recruitment significantly reduced atelectasis formation. The remaining 40% of studies [23,24] focused on the impact of prone positioning on oxygenation, with a duration of 2–4 h being effective for observing changes in PaO_2_/FiO_2_ during the first hour.

No studies reported specific respiratory changes due to pathology or hemodynamic instability. Although prone positioning protocols varied, common practices included facial tilt/rotation and the use of pillows on the chest, abdomen, and extremities to relieve compression. It was suggested that the transition from supine to prone should be managed by an interdisciplinary team in a prospective, randomized controlled trial [24].

In the five included randomized controlled trials, two groups were compared: one assessed invasive mechanical ventilation in the supine position, while the other assessed ventilation in the prone position. Zheng et al. [25] further combined prone positioning with high-frequency oscillatory ventilation, observing improvements in SpO_2_ and PaO_2_ in the prone group compared to the supine group. All studies reported clinical improvements in oxygenation indices (PaO_2_/FiO_2_), lung compliance, mean airway pressure, PaO_2_, and gas exchange with prone positioning. No significant differences were observed, as summarized in Table 2.

## 4. Discussion

This systematic review aimed to assess the protocols and effects of prone positioning during mechanical ventilation in pediatric patients. Five studies were identified, each proposing different protocols; however, a definitive consensus on the transition between supine and prone positions is still lacking. Xu et al. (2021) [27] and Fineman et al. (2006) [28] agree that prior to transitioning from supine to prone, it is essential to evaluate the position of vascular catheters and the endotracheal tube, suspend enteral nutrition 30 min before the transition, and monitor hemodynamics. Diwate et al. (2018) [29] also recommended that pediatric patients remain in the lateral decubitus position for 30 min postprandially as a preventive measure against aspiration.

Sawhney et al. (2005) [24] proposed alternating between the supine and prone positions every 4 h, while Curley et al. (2006) [22] recommended maintaining the prone position for 20 h, followed by 4 h in the supine position. Zheng et al. (2022) [25] combined high-frequency oscillatory ventilation with 12 h of prone positioning in patients with ARDS. This approach resulted in higher SpO_2_ levels and fewer hypoxic episodes, leading to a reduction in FiO_2_ requirements. In contrast, Bruno et al. (2001) [23] studied 18 pediatric patients with an average age of 11 months and found statistically significant differences in SpO_2_ levels between the prone and supine positions (96.2 vs. 98.3, *p* = 0.003), indicating improved oxygenation in the prone position. Jang et al. (2020) [26] reported that ultrasound-guided alveolar recruitment, when combined with the prone position, significantly increased functional residual capacity (FRC) and improved SpO_2_ by 13%, preventing atelectasis in the thoracic regions (47% vs. 8%).

Evidence regarding the effects of prone positioning on pediatric lung function remains limited and controversial. Jang et al. (2020) [26] conducted a randomized, non-controlled study with 18 pediatric patients with severe hypoxemia, ventilated in the prone position for 2 h. After 1 h, five patients showed a significant increase in PaO_2_/FiO_2_, but no significant differences were observed in those with changes in lung function. The efficacy of prone physiotherapy was also evaluated in pediatric patients with V/Q mismatches and compared with conventional physiotherapy. The experimental group received prone physiotherapy for 4 h daily, in 120 min sessions at 6 h intervals. Significant improvements were noted in SpO_2_ (96.2 vs. 93.2) and PaO_2_ (59 mmHg vs. 52 mmHg) levels, with no significant differences in maximum inspiratory pressure (14.60 cmH2O vs. 15.60 cmH2O) [23].

Prone positioning during mechanical ventilation promotes gas exchange and lung function [3,30], but it also carries risks. A randomized controlled trial involving 102 pediatric patients with acute lung injury found no significant reduction in the duration of supplemental oxygenation compared with the supine position (15.6 vs. 15.8 days). The 28-day survival and mechanical ventilation rates were similar, and the mortality rate was 8% in both positions [22]. Furthermore, in the same cohort, adverse effects were noted during prone positioning. While no critical incidents occurred in the 97 patients who were transitioned to the prone position, two cases of endotracheal tube obstruction were observed, and one patient on high-frequency oscillatory ventilation developed hypercapnia [27].

A significant challenge in the current literature on prone positioning for pediatric hypoxemia is the frequent use of small sample sizes, which limits the external validity and generalizability of findings to more heterogeneous pediatric populations. Small cohorts reduce statistical power, hindering the identification of consistent response patterns and rare adverse events, which limit the comprehensive evaluation of the clinical and physiological impacts. Additionally, the lack of standardization in prone positioning protocols complicates the interpretation of research findings [22,23,24,25,26]. Variability in factors such as positioning duration and management strategies introduces inconsistencies across studies. While protocols emphasize patient preparation and continuous monitoring for safety, the absence of standardized guidelines results in practice variability, which affects both outcomes and comparability [16].

The clinical heterogeneity of pediatric hypoxemia further complicates the evaluation of prone positioning. Conditions such as ARDS, infections, congenital malformations, and neuromuscular disorders each involve distinct pathophysiological mechanisms that may alter the response to prone positioning. This makes it difficult to develop a uniform approach applicable to all pediatric subgroups. Furthermore, respiratory physiology and anatomy differ between neonates, infants, and older children, adding another layer of complexity to the assessment of prone positioning. Variations in lung mechanics, respiratory control, and ventilation distribution influence the response to the intervention. These age-related differences necessitate distinct clinical protocols for each subgroup to optimize outcomes [31,32,33].

Despite these variations, prone positioning has been shown to improve oxygenation by increasing the PaO_2_/FiO_2_ ratio and arterial oxygen saturation through better ventilation homogeneity, alveolar recruitment, and reduced cardiac compression on the lung parenchyma [31,32,33]. Improved lung compliance and enhanced regional ventilation further optimize gas exchange. While serious complications such as airway obstruction and hemodynamic instability are infrequent, they do require controlled sedation and preventive measures against pressure injuries [34]. Current evidence supports prone positioning as an effective and safe intervention to improve respiratory function in pediatric patients with hypoxemic respiratory failure [35,36].

In the PICU, the effects of prone mechanical ventilation and the associated protocols have been analyzed to inform clinical decision-making. The lack of standardized protocols regarding the optimal duration of prone positioning underscores the need for further research. Although adapting existing protocols to individual patient conditions is recommended, establishing evidence-based, standardized protocols is essential for improving clinical outcomes.

The reviewed studies show significant variability in patient populations, intervention protocols, and outcome measures. Differences in prone positioning duration (ranging from 2 to 20 h), ventilation modalities, and timing of physiological assessments hinder direct comparisons of efficacy and safety outcomes. Some studies also combined prone positioning with adjunct therapies such as high-frequency oscillatory ventilation or ultrasound-guided alveolar recruitment, while others did not. This variability in methodologies may have influenced the oxygenation and lung mechanics results. Moreover, differences in patient age and severity of hypoxemia further limit the generalizability of the findings.

Despite supporting evidence of the benefits of prone positioning—such as improved pulmonary function and correction of hypoxemia—several limitations must be considered when interpreting the results. Variations in study designs, patient characteristics, and methodological approaches hinder direct comparisons and restrict the applicability of findings. Additionally, the lack of standardized protocols for prone mechanical ventilation in pediatric patients remains a barrier to its routine clinical implementation. These challenges underscore the need for multicenter studies with enhanced methodological rigor and standardized protocols to strengthen the evidence on the safety and efficacy of this intervention in the pediatric population.

## 5. Conclusions

The implementation of prone positioning as a therapeutic strategy in pediatric patients with hypoxemia has shown positive effects on key respiratory parameters, including improvements in oxygenation and ventilatory mechanics. However, challenges remain regarding the standardization of the procedure, inclusion criteria, intervention duration, and monitoring of potential adverse effects. This knowledge gap underscores the importance of continuing to develop controlled, well-structured studies that will allow for the establishment of robust clinical guidelines, tailored to the specific physiological needs of the pediatric population.

## Figures and Tables

**Table 1:** Evaluation of methodological quality according to the PEDro scale.

First Author	1 *	2	3	4	5	6	7	8	9	10	11	Total	Methodological Quality
Curley et al., 2006 [22]	-	1	1	0	1	1	1	1	0	1	0	8	High
Bruno et al., 2001 [23]	-	1	1	1	1	0	1	1	0	1	1	8	High
Sawhney et al., 2005 [24]	-	1	1	1	0	0	0	1	1	1	0	6	Intermediate
Zheng et al., 2022 [25]	-	1	0	0	1	0	0	1	1	1	1	6	Intermediate
Jang et al., 2020 [26]	-	1	1	0	1	0	1	1	1	1	1	8	High

PEDro scale criteria: (1) Eligibility criteria were specified (*—this item is not used in the PEDro score calculation). (2) Subjects were randomly assigned to groups (in crossover studies, subjects were randomized as they received treatments). (3) Allocation was concealed. (4) Groups were similar at baseline concerning important prognostic indicators. (5) All subjects were blinded. (6) All therapists administering the intervention were blinded. (7) All raters assessing at least one key outcome were blinded. (8) Measures for at least one key outcome were obtained from more than 85% of subjects initially assigned to groups. (9) Results were presented for all subjects who received the treatment or were assigned to the control group; when this was not possible, data for at least one key outcome was analyzed on an intention-to-treat basis. (10) Statistical comparisons between groups were reported for at least one key outcome. (11) The study provided point and variability measures for at least one key outcome. Abbreviations: 1 = item met, 0 = item not met. Quality criteria: ≥7 high quality; 5–6 intermediate quality; ≤4 low quality.

**Table 2:** Key characteristics and outcomes of studies on prone positioning in pediatric ventilation.

Author and Year	Country	Sample Size	Age Range	Control Group	Study Design	Cause of PICU Admission	Intervention Protocol	Effects on Oxygenation	Complications	Conclusion
Curley et al., 2006 [22]	USA	102 children	Infants < 3 years	Group 1: Supine position Group 2: Prone position (7 days)	Controlled, randomized, multicenter, non-crossover trial	Patients with acute lung injury	Prone position with individual support (20 h/day, maximum 7 days, transitioning every 20 h)	The incidence of atelectasis was 39.1% lower	Not reported	Prone mechanical ventilation for 7 days supports a multidisciplinary approach in pediatric patients.
Bruno et al., 2001 [23]	Brazil	18 children	Mean age: 11 months	Not applicable	Prospective, non-randomized study	Infants diagnosed with acute respiratory failure	Prone position (6 h ventilation, 2 h prone)	Decreased FiO_2_ requirement.	Increased airway resistance in bronchiolitis patients.	Prone position for at least one hour improves oxygenation in pediatric patients with severe hypoxemia.
Sawhney et al., 2005 [24]	India	42 children	Not reported	Group 1: 21 children treated with mechanical ventilation in prone position Group 2: 21 children treated with mechanical ventilation in supine position	Prospective, randomized, controlled trial	Various pathologies	Transition to prone (4 h in each position, with 2–4 people, securing catheters)	Significant improvement in SpO_2_ and oxygenation index after 5 h.	Not reported	Early prone positioning improves oxygenation and reduces mortality and PVAm.
Zheng et al., 2022 [25]	Not reported	65 children	Infants	Group 1: 33 patients ventilated in supine position Group 2: 32 patients ventilated in prone position	Single-center, prospective, randomized, controlled study	Infants with acute respiratory distress syndrome	Prone position (12 h for 1 day)	Increased PaO_2_/FiO_2_ ratio and reduced duration of invasive mechanical ventilation.	No cardiovascular or pulmonary complications.	HFOV with prone positioning significantly improves oxygenation in infants with ARDS after cardiac surgery.
Jang et al., 2020 [26]	Korea	73 children	<3 years	Experimental group: 37 children ventilated in prone position with recruitment Control group: 36 patients receiving conventional treatment	Prospective, randomized clinical trial	Infants scheduled for elective non-cardiac surgery	Prone position with hourly alveolar recruitment. Control group received conventional treatment.	Significant reduction in atelectasis incidence (22% to 10%).	Not reported	Regular alveolar recruitment reduces atelectasis in children under 3 years undergoing general anesthesia in the prone position.

VOAF-PP: high-frequency oscillatory ventilation combined with prone position; PICU: pediatric intensive care unit; SpO_2_: oxygen saturation; PaO_2_: arterial oxygen pressure; PVAm: mean airway pressure; FiO_2_: inspired fraction of oxygen.

## Abbreviations

The following abbreviations are used in this manuscript:

V/Q	Ventilation–perfusion ratio
ARDS	Acute respiratory distress syndrome
PICUs	Pediatric intensive care units
VOAF-PP	High-frequency oscillatory ventilation combined with prone position
SpO_2_	Oxygen saturation
PaO_2_	Arterial oxygen pressure
PVAm	Mean airway pressure
FiO_2_	Inspired fraction of oxygen
FRC	Functional residual capacity

## Appendix A

Search strategies carried out in each database.

**Database**	**Search Date**	**Search Equation**	**Articles Found**
Scopus	7 January 2025	((((((Pediatric)) OR (Infant)) OR (Child)) AND (Prone)) AND (Protocol)) AND (Respiration, Artificial)	542
PubMed	7 January 2025	((((((Pediatric)) OR (Infant)) OR (Child)) AND (Prone)) AND (Protocol)) AND (Respiration, Artificial)	23
Web of Science	8 January 2025	((((((Pediatric)) OR (Infant)) OR (Child)) AND (Prone)) AND (Protocol)) AND (Respiration, Artificial)	5
ScienceDirect	8 January 2025	((((((Pediatric)) OR (Infant)) OR (Child)) AND (Prone)) AND (Protocol)) AND (Respiration, Artificial)	1463
